# Urinary phosphorus excretion per creatinine clearance as a prognostic marker for progression of chronic kidney disease: a retrospective cohort study

**DOI:** 10.1186/s12882-015-0118-1

**Published:** 2015-07-28

**Authors:** Tomoki Kawasaki, Yoshitaka Maeda, Hisazumi Matsuki, Yuko Matsumoto, Masanobu Akazawa, Tamaki Kuyama

**Affiliations:** Nephrology Division, Department of Internal Medicine, JA Toride Medical Center, Ibaraki, Japan

**Keywords:** Collected urine, Chronic kidney diseases, End-stage kidney disease, Bone diseases, Metabolic, Protein intake

## Abstract

**Background:**

Whether phosphate itself has nephrotoxicity in patients with chronic kidney disease (CKD) is controversial, although phosphate excretion into urine may cause tubular damage in rat models. To evaluate actual phosphate load on each nephron, we examined the association between 24-h urinary phosphorus excretion per creatinine clearance (24-h U-P/C_Cr_), a newly proposed index that is a surrogate for nephron load, and CKD progression in patients with CKD.

**Methods:**

We conducted a single-center, retrospective cohort study. To avoid potential confounders for protein intake, only patients on our educational program for CKD with a fixed diet regimen and aged 20 years or older were included. The observation period was 3 years. Primary outcomes were CKD progression defined as a composite of end-stage kidney disease (ESKD) or 50 % reduction of estimated glomerular filtration rate. Patients were stratified by quartiles of 24-h U-P/C_Cr_ levels as Quartiles 1–4. The association was examined in three models: unadjusted (Model 1), adjusted for risk factors for CKD progression (Model 2), and factors that affect renal phosphate handling (Model 3).

**Results:**

A total of 191 patients met the eligibility criteria. Patients with higher 24-h U-P/C_Cr_ showed a higher risk for the composite outcomes. The hazard ratios [95 % confidence interval] for 24-h U-P/C_Cr_ levels in Quartile 2, 3, and 4, respectively, versus Quartile 1 were 2.56 (1.15–6.24), 7.53 (3.63–17.62), and 12.17 (5.82–28.64) in Model 1; 1.66 (0.63–4.97), 3.57 (1.25–11.71), and 5.34 (1.41–22.32) in Model 2; and 3.07 (0.97–11.85), 7.52 (2.13–32.69), and 7.89 (1.74–44.33) in Model 3.

**Conclusions:**

Our study showed that higher phosphorus excretion per creatinine clearance was associated with CKD progression.

## Background

Serum phosphorus level is a risk factor for cardiovascular disease in patients with chronic kidney disease (CKD) [1–3]. Several studies also reported that higher serum phosphorus levels were associated with CKD progression [4, 5]. Meanwhile, there is considerable evidence that serum phosphorus level is dependent on protein intake [6]. Renal phosphate handling is affected by phosphate loading and depletion, parathyroid hormone, 1,25-dihydroxyvitamin D (1,25(OH)VitD)‚ volume, hyper- and hypocalcemia, glucose, acid–base disturbance, dopamine, and fibroblast growth factor (FGF)-23 [7]. Of these, parathyroid hormone and FGF-23 have been considered as especially important factors that regulate renal phosphate handling [8]. Although the mechanism between high serum phosphorus and CKD progression is not fully elucidated, it is assumed that phosphate excretion into urine causes tubular damage. The degree of kidney damage depends not only on the amount of phosphate load but also the nephron number [9], while tubular damage occurred in rats when phosphate excretion per nephron exceeded 1 μg/day [10]. To evaluate actual phosphate burden on residual nephrons, we proposed a new index, 24-h urinary phosphorus excretion per creatinine clearance (24-h U-P/C_Cr_), as a prognostic factor for CKD progression. In animal studies, renal biopsy and magnetic resonance imaging were used to estimate glomerular number. A previous study reported a positive correlation between glomerular filtration rate (GFR) and glomerular number in stable renal transplants [11]. Thus, we used 24-h C_Cr_ as a marker for nephron number. To avoid potential confounders for protein intake, only patients on the educational program for CKD with a fixed diet regimen were included. The association between 24-h U-P/C_Cr_ and CKD progression in these patients was examined.

## Methods

### Study population and outcomes

We conducted a single-center, retrospective cohort study in a Japanese population to examine the association between 24-h U-P/C_Cr_ and CKD progression.

Patients aged 20 years and older who were admitted to the hospital and who were in the educational program for CKD between January 2001 and December 2006 were enrolled. A fixed amount of protein (0.6 to 0.8 g/kg, standard body weight) was served to each patient during the whole admission period. The proportion of animal protein in the diet regimen was 50–60 % of the total. Therefore, the phosphate intake of each patient was also fixed at about 0.8 to 1.0 g/kg of standard body weight. Patients with acute kidney injury or primary hyperparathyroidism were excluded. Although the observation period was 3 years, patients who were lost to follow-up were censored at the date of the last contact with an attending physician of the hospital.

Data on age, weight, and sex of the patients, and comorbid conditions, including history of diabetes mellitus and laboratory data, were obtained by review of medical records. Laboratory data included serum creatinine (mg/dL), urea nitrogen (mg/dL), albumin (g/dL), calcium (mg/dL), phosphorus (mg/dL), intact parathormone (PTH) (pg/mL), 1,25(OH)VitD (pg/mL), C_Cr_ (ml/min), 24-h urea nitrogen excretion (g/day), 24-h urinary protein (g/day) and 24-h urinary phosphorus (mg/day). Serum calcium concentration was corrected for serum albumin concentration using the following formula: Corrected calcium = measured calcium − (4 − serum albumin) [12]. Estimated GFR (eGFR) was calculated using the equation developed by the Japanese Society of Nephrology to adjust for characteristics of Japanese patients as follows: eGFR = 194 × serum creatine^−1.094^ × age^−0.287^ (if female, × 0.739) [13]. Daily protein intake was estimated using Maroni’s formula as follows: Estimated protein intake = (urea nitrogen excretion [g/day] + 0.031 [g/kg] × body weight [kg]) × 6.25) [14]. Because over 50 % of patients had no record about use of angiotensin receptor blockers, angiotensin-converting enzyme inhibitors or other drugs, or history of hypertension and heart disease or smoking status, we did not include these factors as variables.

**Fig. 1:**
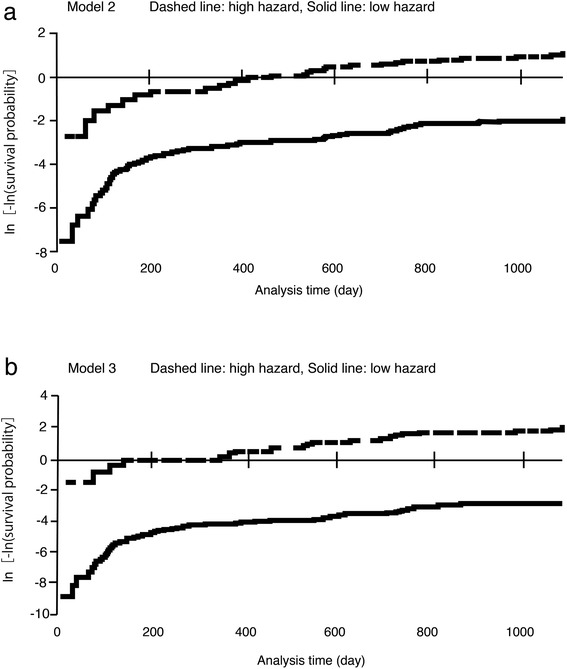
Proportional hazards with the applied predictors in the Cox model were assessed by plotting a negative logarithm of the Kaplan–Meier Survivor estimate. The hazard ratio in each patient was calculated, and then patients were divided into two groups according to the median of the hazard ratio. **a** Proportionality of hazards in Model 2 divided into high and low hazard. **b** Proportionality of hazards in Model 3, as in (**b**)

The primary outcomes of this study were CKD progression defined as a composite of end-stage kidney disease (ESKD) or 50 % reduction in eGFR. We compared subgroups stratified by quartile of 24-h U-P/C_Cr_ levels: ≤ 11.15 mg/day/C_Cr_ (Quartile 1), 11.16 to 17.07 mg/day/C_Cr_ (Quartile 2), 17.08 to 29.61 mg/day/C_Cr_ (Quartile 3), and ≥ 29.62 mg/day/C_Cr_ (Quartile 4). We also examined the association between 24-h U-P/C_Cr_ and serum phosphorus. Associations were examined in models with incremental multivariable adjustments: unadjusted (Model 1); comorbid conditions, and risk factors for CKD progression such as age, male sex, diabetes, C_Cr_, protein intake, urinary protein, and corrected calcium (Model 2); and factors that influence phosphate reabsorption such as age, male sex, C_Cr_, protein intake, corrected calcium, 1,25(OH)VitD, and PTH (Model 3). Unadjusted and multivariable-adjusted relative risks for progressive CKD in relation to 24-h U-P/C_Cr_ were calculated using Cox proportional hazard models. The assumption of proportional hazards in the Cox regression models was assessed and found to be acceptable (Fig. 1).

To compare 24-h U-P/C_Cr_ with common markers of urinary phosphorus excretion, we examined for the association of fractional excretion of phosphorus (FeP), tubular reabsorption of phosphate (TRP), and maximum tubular reabsorption of phosphorus per unit volume of glomerular filtration rate (TmP/GFR) with primary outcomes. TRP was calculated using the following formula: TRP = 1 − (urinary phosphate × serum creatinine)/(serum phosphate × urinary creatinine). We also analyzed an alternate model, where 24-h U-P/eGFR, instead of 24-h U-P/C_Cr_, was applied to a variable, because this is more commonly used by Japanese physicians.

A total of 191 patients met the eligibility criteria. Of these, one (0.5 %) patient had primary hyperparathyroidism, eight (4 %) patients had evidence of acute kidney injury and seven (3 %) patients had missing values, excluding them from analyses. 1,25(OH)VitD or PTH were missing in 24 (15 %) patients, excluding them from analyses only in Model 3. The final analyses were carried out in 175 patients in Model 1 and Model 2, and 151 patients in Model 3. Statistical analyses were performed using JMP, version 11.2, (SAS Institute Inc., Cary, NC, USA), and Excel-Toukei 2012 (Social Survey Research Information Co., Ltd., Japan), where *p* < 0.05 was considered statistically significant.

### Ethics

This study was approved by the ethics committee of JA Toride Medical Center, and the research was performed in accordance with the ethical principles of the Declaration of Helsinki. According to the ethics committee of JA Toride Medical Center, there was no need for patient consent for this anonymous retrospective study.

## Results

**Fig. 2:**
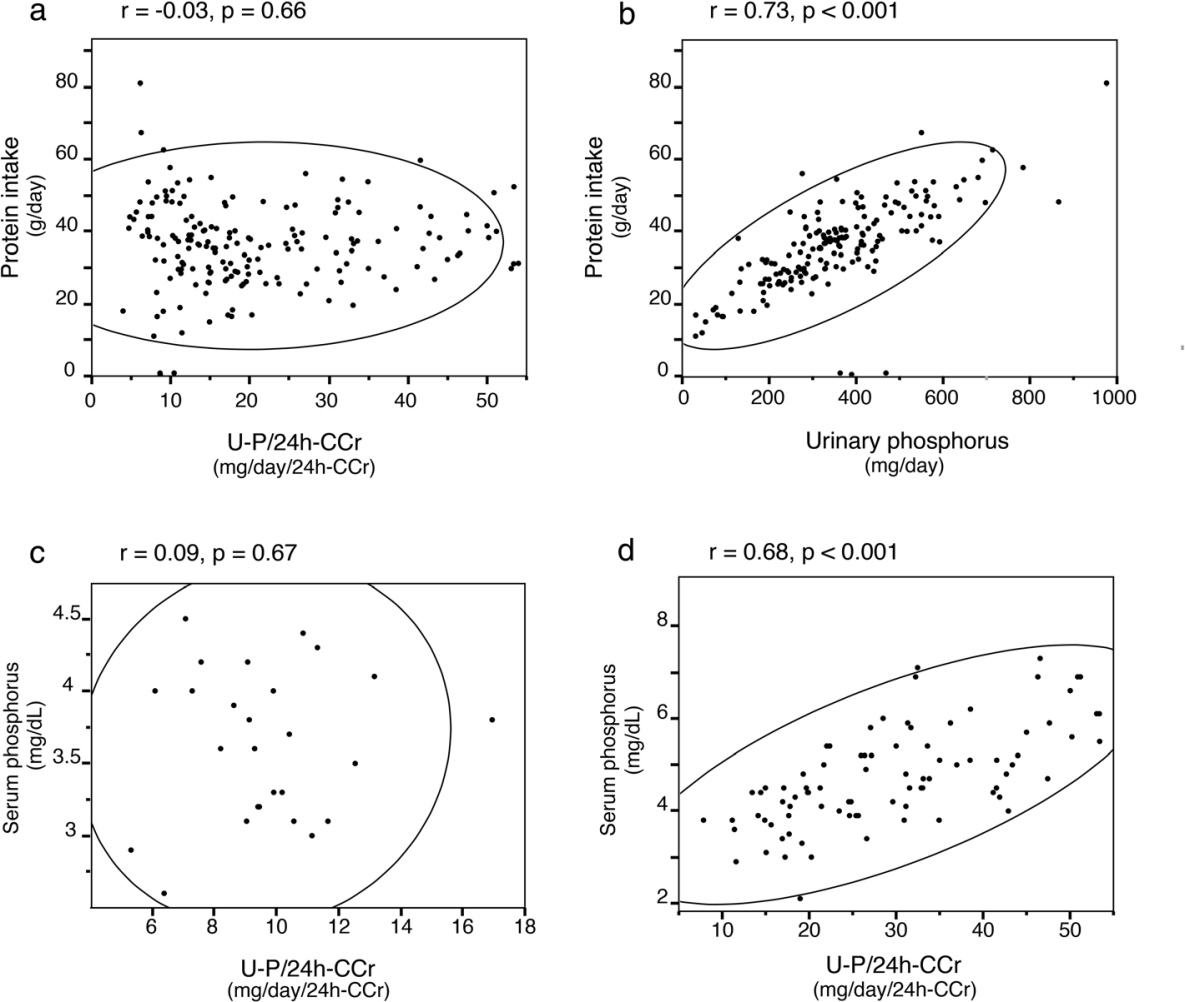
Bivariate fit plot. **a** Bivariate fit plot of protein intake by 24-h urinary phosphorus per creatinine clearance. **b** Bivariate fit plot of protein intake by urinary phosphorus. **c** Bivariate fit plot of serum phosphorus by urinary phosphorus per 24-h creatinine clearance (24-h U-P/C_Cr_) in patients with CKD stage G3b. **d** Patients with CKD stage G5. r denotes Pearson’s correlation coefficient, p denotes p values for correlation, and the ellipsoidal line indicates Bivariate Normal Ellipse where *p* = 0.95

**Fig. 3:**
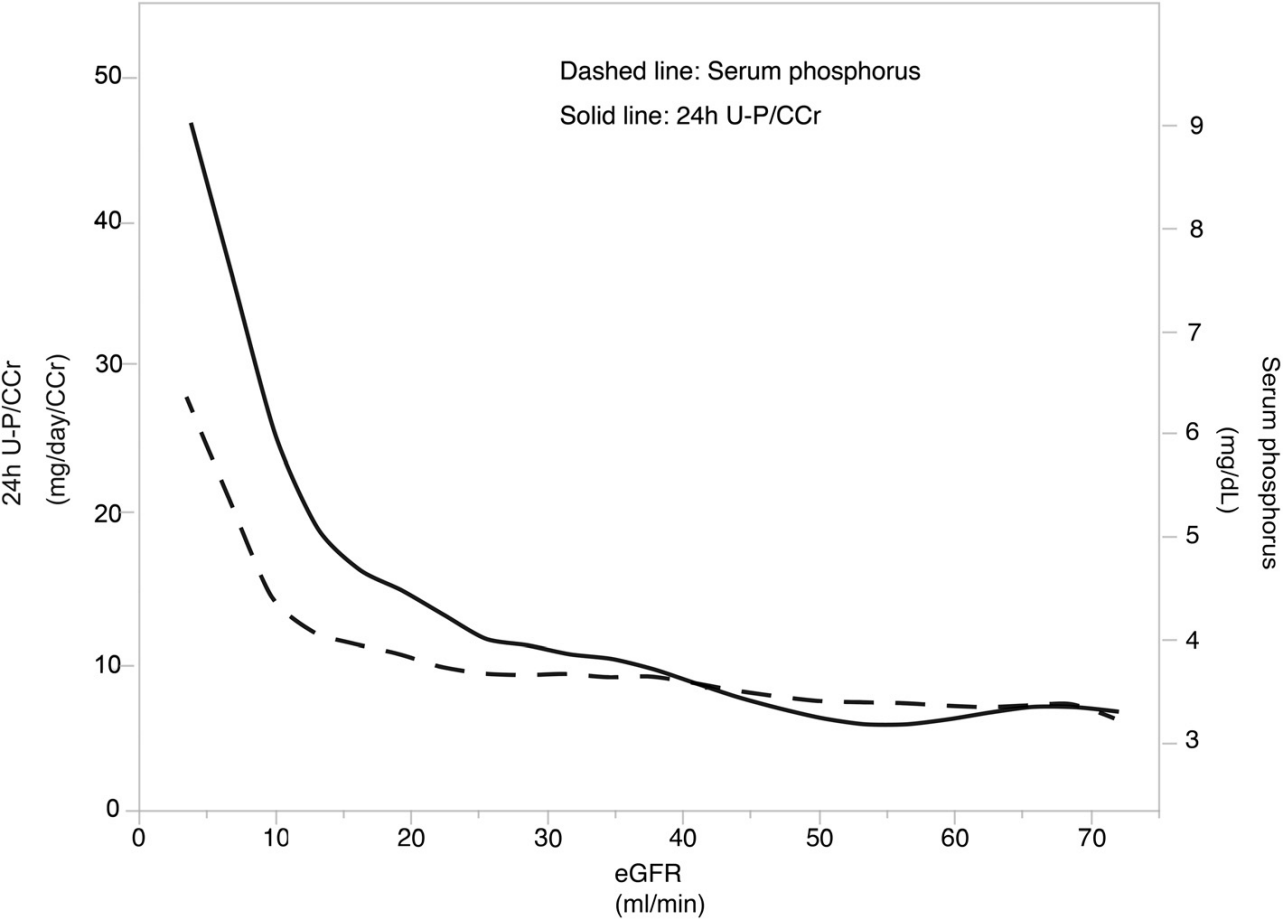
Association between urinary and serum phosphorus and eGFR. 24-h U-P/C_Cr_, urinary phosphorus per 24-h creatinine clearance. 24-h U-P/C_Cr_ denotes urinary phosphorus per 24-h creatinine clearance

Baseline characteristics are shown in Table 1 for patient groups stratified by 24-h U-P/C_Cr_ quartile. Mean age of the studied population was 65 years, and 69 % were male. Prevalence of diabetes mellitus was 34 %. Mean eGFR was 19.1 ml/min/1.73 m^2^. The patients with CKD stage G5 represented the largest population and there was no patient with CKD stage G1. Patients with Quartile 3 had lower protein intake, but when 24-h U-P/C_Cr_ was included as a continuous variable, a significant statistical correlation was not observed between 24-h U-P/C_Cr_ and protein intake (Fig. 2a). Pearson’s correlation coefficient (r) for this correlation was −0.03 (*p* = 0.66). In contrast, a highly significant statistical correlation between urinary phosphorus and protein intake was observed (*r* = 0.73, *p* < 0.001) (Fig. 2b). Fig. 3 shows that 24-h U-P/C_Cr_ was elevated earlier than serum phosphorus in CKD patients. Patients in Quartile 4 had lower eGFR and corrected calcium and higher serum phosphorus, PTH and urinary protein.

**Table 1:** Baseline characteristics stratified by quartiles of urinary phosphorus per creatinine clearance

	All	Quartile 1	Quartile 2	Quartile 3	Quartile 4	*P* Value
	(*N* = 175)	≤ 11.15	11.16–17.07	17.08–29.61	≥ 29.62	
		(*N* = 44)	(*N* = 44)	(*N* = 43)	(*N* = 44)	
Age	65.4 ± 12.3	67.4 ± 14.1	66.9 ± 11.0	66.9 ± 9.64	61.1 ± 13.1	0.07
Male sex (n, %)	120 (68.5)	35 (79.6)	31 (70.5)	26 (60.5)	28 (63.6)	0.21
Body mass index	23.6 ± 3.58	23.2 ± 3.82	23.7 ± 3.75	23.1 ± 3.64	24.2 ± 3.07	0.44
Diabetes mellitus (n, %)	59 (33.7)	15 (34.1)	14 (32.6)	18 (32.6)	18 (40.9)	0.60
Protein intake (g/day)	36.8 ± 10.78	41.1 ± 13.7	35.0 ± 8.97	33.0 ± 9.00	37.9 ± 9.24	0.10
Cr (mg/dL)	3.73 ± 2.22	1.70 ± 0.56	2.68 ± 1.01	3.83 ± 1.13	6.71 ± 1.70	< 0.001
estimated GFR (ml/min/1.73 m2)	19.1 ± 13.5	35.3 ± 14.3	20.7 ± 7.34	13.0 ± 4.18	7.34 ± 2.77	< 0.001
CKD stage G2 (n,%)	4 (2.3)	4 (9.1)	0 (0.0)	0 (0.0)	0 (0.0)	
G3a (n,%)	4 (2.3)	4 (9.1)	0 (0.0)	0 (0.0)	0 (0.0)	
G3b (n,%)	26 (14.9)	21 (47.7)	5 (11.4)	0 (0.0)	0 (0.0)	
G4 (n,%)	55 (31.4)	13 (29.5)	27 (61.4)	13 (30.2)	2 (45.5)	
G5 (n, %)	86 (49.1)	2 (4.5)	12 (27.3)	30 (69.8)	42 (95.5)	
24-h-CCr (ml/min)	24.7 ± 22.1	49.4 ± 28.7	24.7 ± 12.1	14.6 ± 5.93	9.83 ± 3.66	< 0.001
Corrected Ca (mg/dL)	9.41 ± 0.63	9.72 ± 0.44	9.57 ± 0.51	9.39 ± 0.51	8.96 ± 0.75	< 0.001
Serum phosphorus (mg/dL)	4.20 ± 1.07	3.50 ± 0.57	3.76 ± 0.58	4.19 ± 0.82	5.35 ± 1.14	< 0.001
iPTH (pg/mL)	139.0 ± 134.1	54.8 ± 27.8	95.7 ± 52.2	156.3 ± 114.3	261.5 ± 186.6	< 0.001
1,25(OH)VitD (pg/mL)	24.7 ± 22.1	36.9 ± 16.9	26.1 ± 10.9	22.0 ± 9.9	13.0 ± 6.1	< 0.001
Urinary protein (g/day)	2.15 ± 2.46	1.18 ± 1.98	1.86 ± 2.03	1.94 ± 1.93	3.63 ± 3.07	< 0.001
Urinary phosphorus (mg/day)	356 ± 158	393 ± 201	342 ± 163	310 ± 114	379 ± 129	0.06

Data are means ± SD or n (% of total), Comparisons were made by *χ* ^2^ test or analysis of variance (ANOVA). Continuous variables were expressed as mean ± standard deviation (SD), and categorical variables were expressed as proportions. Categorical variables were tested by *χ* ^2^ test, and continuous variables were compared by ANOVA. The homogeneity of the variances was analyzed by the Leven test; in those cases in which the variances were unequal, the variables were compared by Welch’s test. Cr, serum creatinine levels; GFR, glomerular filtration rate; 24-h-C_Cr_, 24-h creatinine clearance; Ca, calcium; iPTH, intact parathormone; 1,25(OH)VitD, 1,25 dihydroxyvitaminD

The mean 24-h U-P/C_Cr_ was 20.9 ± 12.7 mg/day/C_Cr_ and the median value was 17.07 mg/day/C_Cr_. The maximum and minimum values were 53.97 mg/day/C_Cr_ and 3.95 mg/day/C_Cr_, respectively. The first quartile value was 11.15 mg/day/C_Cr_, and the third quartile was 29.61 mg/day/C_Cr_. A significant correlation was observed between serum phosphorus and U-P/C_Cr_ in patients with CKD stage G5 (*r* = 0.68, *p* < 0.001), but not in patients with CKD stage G3b (*r* = 0.09, *p* = 0.67) as shown in Fig. 2c and d.

Median follow-up period was 1.56 year. Ninety (51 %) patients reached the composite outcomes of ESKD or 50 % reduction of eGFR over 3 years, of whom 50 (29 %) reached ESKD and 40 (23 %) had a greater than 50 % reduction. More patients in Quartile 4 reached the composite outcomes (Table 2).

**Table 2:** Distribution of events by quartiles of phosphorus excretion per creatinine clearance

	Composite end point	ESKD	50 % reduction of eGFR
All	90	50	40
Quartile 1	8 (8.9)	1 (2.0)	7 (17.5)
Quartile 2	18 (20.0)	5 (10.0)	13 (32.5)
Quartile 3	32 (35.6)	14 (28.0)	18 (45.0)
Quartile 4	32 (35.6)	30 (60.0)	2 (5.0)

Data are depicted as number of patients (% of total)

*ESKD* end-stage kidney disease, *eGFR* estimated glomerular filtration rate

The hazard ratio (HR) and 95 % confidence interval (CI) of the composite outcomes in the unadjusted Cox proportional hazard model and after adjustment are shown in Table 3. Higher 24-h U-P/C_Cr_ was associated with a higher risk for the composite outcomes in all models. In Model 1, the HRs and 95 % CIs for Quartile 2, Quartile 3, and Quartile 4, respectively, with Quartile 1 as a reference were 2.56 (1.15–6.24), 7.53 (3.63–17.62), and 12.17 (5.82–28.64). The corresponding values were 1.66 (0.63–4.97), 3.57 (1.25–11.71), and 5.34 (1.41–22.32) in Model 2, and 3.07 (0.97–11.85), 7.52 (2.13–32.69), and 7.89 (1.74–44.3) in Model 3. The alternate model, where 24-h U-P/eGFR, instead of 24-h U-P/C_Cr_, was applied to a variable, showed similar results (Table 4). TRP and eGFR were correlated (*r* = 0.68, *p* < 0.001). However, neither TRP nor TmP/GFR were associated with the composite outcomes (data not shown). Moreover, the association between FeP and the outcome was observed in Model 1, while in Models 2 and 3, higher FeP was not associated with a higher risk for the outcomes.

**Table 3:** Hazard ratio (HR) and 95 % confidence interval (CI) of the composite outcome of end-stage kidney disease or 50 % reduction of estimated glomerular filtration rate associated with urinary phosphorus excretion

	Model 1		Model 2		Model 3	
	HR (95%CI)	P Value	HR (95%CI)	P Value	HR (95%CI)	P Value
Quartile 1	1.00		1.00		1.00	
	(reference)		(reference)		(reference)	
Quartile 2	2.56	0.02	1.66	0.31	3.07	0.057
	(1.15–6.24)		(0.63–4.97)		(0.97–11.85)	
Quartile 3	7.53	< 0.001	3.57	0.01	7.52	0.001
	(3.63–17.62)		(1.25–11.71)		(2.13–32.69)	
Quartile 4	12.17	< 0.001	5.34	0.01	7.89	0.006
	(5.82–28.64)		(1.41–22.32)		(1.74–44.33)	

Quartile 1 served as a reference. Model 2: Adjusted for age, male sex, diabetes mellitus, 24-h creatinine clearance, protein intake, urinary protein, corrected calcium. Model 3: Adjusted for age, male sex, 24-h creatinine clearance, protein intake, corrected calcium, 1,25-dihydroxyvitamin D and intact parathormone

**Table 4:** Hazard ratio (HR) and 95 % confidence interval (CI) of the composite outcome of end-stage kidney disease or 50 % reduction of estimated glomerular filtration rate (eGFR) associated with urinary phosphorus excretion in an alternative model, where urinary phosphorus excretion per eGFR was applied to a variable

	Model 1		Model 2		Model 3	
	HR (95 % CI)	P Value	HR (95 % CI)	P Value	HR (95 % CI)	P Value
eGFR	1.00		1.00		1.00	
Quartile 1	(reference)		(reference)		(reference)	
eGFR	1.31	0.44	1.85	0.10	1.38	0.42
Quartile 2	(0.65–2.72)		(0.87–4.04)		(0.63–3.17)	
eGFR	2.52	0.004	2.74	0.01	2.37	0.03
Quartile 3	(1.32–5.06)		(1.27–6.15)		(1.06–5.58)	
eGFR	5.62	< 0.001	3.98	0.005	2.58	0.05
Quartile 4	(3.00–11.14)		(1.51–10.33)		(0.97–6.92)	

Quartile 1 served as a reference. Model 2: Adjusted for age, male sex, diabetes mellitus, 24-h creatinine clearance, protein intake, urinary protein, corrected calcium. Model 3: Adjusted for age, male sex, 24-h creatinine clearance, protein intake, corrected calcium, 1,25-dihydroxyvitamin D and intact parathormone

## Discussion

Our study showed that higher phosphorus excretion per creatinine clearance was associated with CKD progression. This association remained after adjustment for other possible risk factors related to CKD progression.

In a previous animal experiment, phosphate excretion per nephron over 1 μg/day caused tubular damage [10] while in another study, sevelamer hydrochloride, a phosphate binder, was renoprotective in rats [15]. In human studies, high serum phosphorus was reported to be a risk factor for CKD progression [4, 13, 16, 17], while urinary phosphorus excretion has not been studied. This is the first report to look for a possible association between urinary phosphorus excretion and CKD progression, although the underlying mechanism between high phosphate excretion and CKD progression has not been fully elucidated. High urine phosphorus may cause renal tubular damage and renal fibrosis by forming calcium phosphate crystals via oxidative stress [18]. A recent cross-sectional analysis showed that TRP was associated with eGFR [19]. In our study, TRP was associated with eGFR, as previous studies reported [19, 20], but did not show any relation to CKD progression. A previous study showed no impact of FeP on CKD progression [21]. In this study, patients were categorized according to FGF-23 and FeP values. In our study, there was no adjustment for FGF-23 because 24-h U-P/C_Cr_ would be a confounding factor for FGF-23. FGF23 cannot be measured in clinical use in Japan so U-P/24 h-C_Cr_ may be a suitable surrogate marker.

U-P/24-h-C_Cr_ would be a confounding factor for FGF23. A significant correlation was observed between serum phosphorus and U-P/24-h-C_Cr_ in patients with CKD stage G5, but not those with CKD stage G3b. In early stage CKD, serum phosphorus was reported to remain in the normal range due to an increase in phosphorus excretion by FGF-23 [9, 22]. In addition, further studies reported that elevated serum FGF-23 was associated with a decline in eGFR [23]. Therefore, early intervention for phosphate restriction by lowering FGF-23 but not serum phosphorus levels was suggested as a new strategy against CKD progression [9, 24, 25]. In such cases, urinary phosphorus excretion per creatinine clearance could be a useful marker for early intervention for phosphate restriction because of its inexpensive and minimally invasive features.

We applied 24-h C_Cr_ as a marker for nephron numbers, but eGFR is more commonly used by physicians. Therefore, we also examined U-P/eGFR, instead of U-P/24-h-C_Cr_, as a marker for phosphorus burden to nephrons, and found similar reliability.

24-h U-P/C_Cr_ may be superior to 24-h U-P/eGFR for the following reason. It is difficult to collect 24-h urine accurately because of residual urine. The units of U-P/24-h C_Cr_ and U-P/eGFR are presented as follows: (mg/24 h)/(ml/min/24 h) and (mg/24 h)/(ml/min). If collected urine is sampled only over 23 h, the value of U-P/24-h C_Cr_ does not change greatly, because U-P and 24-h C_Cr_ were collected during the same time. In this case, the unit of U-P/24-h C_Cr_ is (mg/23 h)/(ml/min/23 h). However, when normalizing eGFR, each urine sample must be collected accurately over 24 h.

### Study strengths and limitations

The strength of our study is that we included protein intake as a potential confounding factor. As a direct effect of phosphate on the kidney, oral phosphate overload damaged the kidney while oral phosphate restriction prevented CKD progression in animals [26, 27]. However, it is certain that urinary phosphate is a surrogate marker reflecting protein intake that is associated with CKD progression [28]. Indeed, protein intake was correlated with urinary phosphorus excretion in our study, but not with 24-h U-P/C_Cr_, which showed that 24-h U-P/C_Cr_ is a risk factor independent of protein intake.

Another strength of our study was using 24-h collected urine samples to measure urinary phosphorus excretion. Compared with spot urine, measuring 24-h collected urine samples takes much time and effort. In CKD patients, the circadian rhythm of urinary phosphorus excretion along with U-P/Cr/eGFR remains unclear, although urinary phosphorus excretion increases from morning to the middle of the night in healthy subjects [29].

Using 24-h collected urine can ensure more accurate measurement of phosphorus excretion than spot urine. However, spot urine could be an alternative prognostic marker if the circadian change of U-P/Cr was resolved.

Our study has several limitations. First, it is a single-center, retrospective observational study, and not an interventional study, in a Japanese population. Second, we mitigated the effect of confounding factors by using multivariate Cox proportional hazard analysis. We applied PTH and 1,25(OH)VitD to the confounding variables in Model 3, but the sample size in each group may have been insufficient for full evaluation.

Third, we assumed the number of nephrons was proportionate to 24-h C_Cr_, but the number of nephrons was not fully proportionate to creatinine clearance. However, a reliable marker for residual nephrons is not available at present. Finally, we measured daily urinary phosphorus excretion only once even under the fixed menu in admitted patients. Day to day variability in urinary phosphorus excretion was not studied.

## Conclusions

Our study showed that higher 24-h U-P/C_Cr_ was associated with more rapid CKD progression. Because an increase of urinary phosphate excretion is reported to be earlier than an elevation in serum phosphorus levels, 24-h U-P/C_Cr_ may be a useful marker for suggesting phosphate burden to each nephron unit, thus enabling earlier interventions. Further study is needed to resolve the mechanism of 24-h U-P/C_Cr_ as a risk factor for CKD progression.

## Abbreviations

1,25(OH)VitD: 1,25-dihydroxyvitamin D
CI: Confidence interval
CKD: Chronic kidney disease
eGFR: Estimated glomerular filtration rate
ESKD: End-stage kidney disease
FGF-23: Fibroblast growth factor-23
HR: Hazard ratio
PTH: Parathormone
TmP/GFR: Tubular reabsorption of phosphorus per unit volume of glomerular filtration rate
TRP: Tubular reabsorption phosphate
24-h U-P/C_Cr_: 24-h urinary phosphorus excretion per creatinine clearance
